# Diagnosis of Tinnitus Due to Auditory Radiation Injury Following Whiplash Injury: A Case Study

**DOI:** 10.3390/diagnostics10010019

**Published:** 2019-12-30

**Authors:** Sung Jun Lee, Chang Hoon Bae, Jeong Pyo Seo, Sung Ho Jang

**Affiliations:** 1 Department of Physical Medicine and Rehabilitation, College of Medicine, Yeungnam University, 317-1, Daemyungdong, Namku, Taegu 705-717, Korea; hssj8020@hanmail.net; 2 Department of Otorhinolaryngology-Head and Neck Surgery, College of Medicine, Yeungnam University, Daegu KS002, Korea; baich@med.yu.ac.kr; 3 Department of Physical Therapy, College of Health Sciences, Dankook University, Cheonan KS002, Korea; raphael0905@hanmail.net

**Keywords:** diffusion tensor tractography, tinnitus, auditory radiation, traumatic axonal injury, whiplash injury

## Abstract

We report on a patient with tinnitus who showed injury of auditory radiation following whiplash injury, demonstrated by diffusion tensor tractography (DTT). A 48-year-old male patient suffered from a car crash resulting in flexion-hyperextension injury of his head after being hit from behind by a moving car while waiting at a signal while driving a car. Three days after the car crash, he began to feel tinnitus in both ears and his tinnitus became aggravated with the passage of time. No specific lesion was observed on a conventional brain MRI performed two weeks after the car crash. Although he visited several hospitals, the precise cause of his tinnitus was not detected. Two years after the car crash, he underwent evaluation for his tinnitus at the ear, nose and throat department of a university hospital. The pure tone audiometry was evaluated in a sound-proof room to screen his hearing status for the frequencies of 250–8000 Hz and no specific abnormality was detected. Although he was also tested for speech audiometry, there was also no specific abnormality. In order to assess his tinnitus, a tinnitogram was conducted to evaluate the frequency content and the loudness. His tinnitus was characterized at an intensity of 40 dB and a frequency of 4000 Hz. However, no abnormality was observed in either ear on physical examination. On DTT, the auditory radiation showed severe narrowing and tearing in both hemispheres. To summarize, neural injury of the auditory radiation was demonstrated in a patient with tinnitus following whiplash injury, using DTT.

## 1. Introduction

Whiplash injury is caused by sudden acceleration–deceleration, especially to the head and neck [1,2]. Approximately 10% of patients who have suffered whiplash injury develop otological symptoms such as tinnitus, deafness, and vertigo [3,4,5,6]. Tinnitus is defined as sounds perceived by the patient in the absence of external auditory stimulation [7]. Although several hypotheses have been suggested on the pathophysiology of trauma-associated tinnitus, altered somatosensory input to the auditory pathways after head or neck injuries has been considered as a plausible pathophysiology [7,8]. By contrast, some researchers have suggested that tinnitus may be related to neural injury of the auditory pathways in the brain [9,10]. However, it has not been clearly elucidated.

After the introduction of diffusion tensor imaging (DTI), many studies using diffusion tensor tractography (DTT), which is reconstructed from DTI data, have demonstrated neural injuries of various neural tracts in patients with cerebral concussion or mild traumatic brain injury (TBI) who showed no abnormal lesion on conventional brain MRI [11,12,13,14,15]. Among these studies, several studies have reported injury of neural tracts and their relavant clinical symptoms following whiplash injury [16,17,18,19]. These neural tracts comprise the corticospinal tract, the corticoreticulospinal tract, the dentatorubrothalamic tract, the ascending reticular activating system, and the spinothalamic tract [16,17,18,19]. Regarding auditory radiation, although a case study has been reported, which demonstrated the relation between sensorineural hearing loss and auditory radiation injury, no study on tinnitus related to auditory radiation injury has been reported [20].

In this study, we report on a patient with whiplash injury involving auditory radiation, which was complicated by a tinnitus, demonstrated by DTT.

## 2. Case Report

A 48-year-old male patient suffered from a car crash resulting in flexion-hyperextension injury of his head after being hit from behind by a moving car while waiting at a signal while driving a car. At the time of the car crash, he did not experience loss of consciousness or post-traumatic amnesia. The patient’s Glasgow Coma Scale score was 15. However, he was diagnosed with subarachnoid hemorrhage and underwent conservative management at a local hospital. Three days after the car crash, he began to feel tinnitus in both ears and his tinnitus became aggravated with the passage of time. No specific lesion was observed on a conventional brain MRI performed two weeks after the car crash. Although he visited several hospitals, the precise cause of his tinnitus was not detected. Two years after the car crash, he underwent evaluation for his tinnitus at the ear, nose and throat department of a university hospital. The pure tone audiometry was evaluated in a sound-proof room to screen his hearing status for frequencies of 250–8000 Hz and no specific abnormality was detected [21]. Although he was also tested for speech audiometry which is a clinical examination that is commonly used to assess the impact of a potential hearing deficit on speech understanding, there was no specific abnormality. In order to assess his tinnitus, a tinnitogram was conducted to evaluate the frequency content and the loudness [21]. His tinnitus was characterized at an intensity of 40 dB and a frequency of 4000 Hz. However, no abnormality was observed in either ear on physical examination. The patient provided written informed consent, and the study protocol was approved by the local Institutional Research Board.

### Diffusion Tensor Tractography

**Figure 1:**
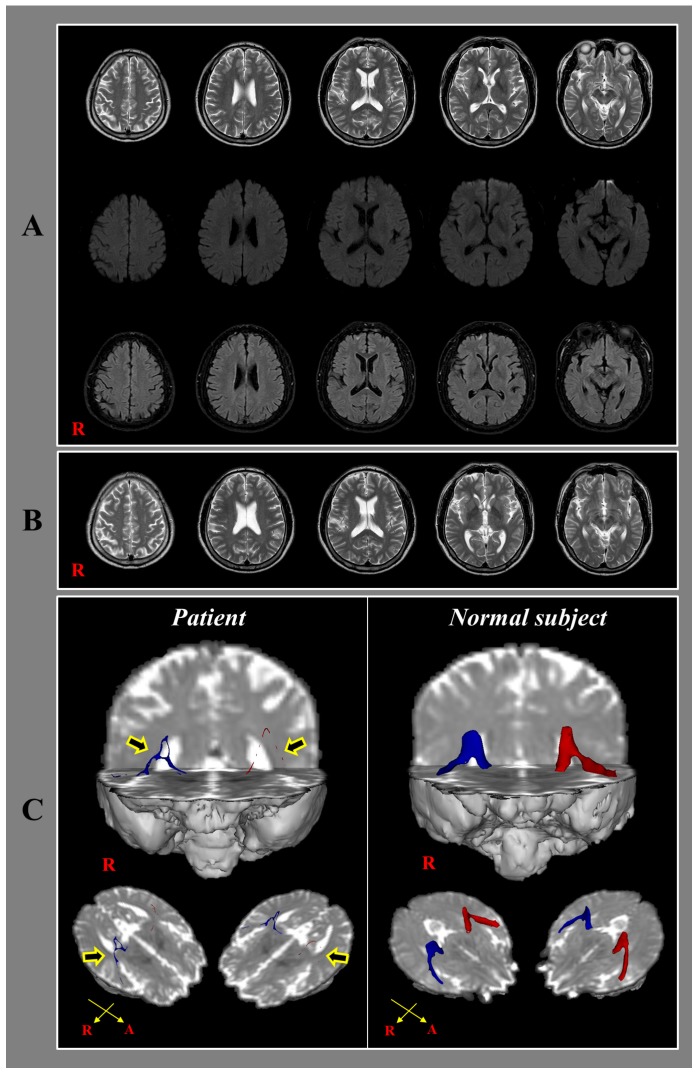
(**A**) T2-weighted (**upper** row), diffusion weighted (**middle** row) and fluid-attenuated inversion recovery images (**lower** row) taken 2 weeks after onset reveals no abnormal lesion; (**B**) T2-weighted images taken 2.5 years after onset shows no abnormal lesion; (**C**) 2.5-year diffusion tensor tractography for the auditory radiation in the patient shows severe narrowing and tearing (arrows) in both hemispheres compared with a normal subject (47-year old female).

Using a 6-channel head coil on a 1.5 T Philips Gyroscan Intera (Philips, Ltd., Best, The Netherlands) with 32 non-collinear diffusion sensitizing gradients by single-shot echo-planar imaging, DTI data were acquired 2.5 years after the car crash. Imaging parameters were as follows: TR: 10,398 ms, TE: 72 ms, *b*: 1000 s/mm^2^, NEX = 1, acquisition matrix: 96 × 96, reconstructed to matrix: 192 × 192 matrix, field of view: 240 × 240 mm^2^, parallel imaging reduction factor (SENSE factor): 2, EPI factor: 59, slice gap = 0, and a slice thickness of 2.5 mm. For analysis of diffusion-weighted imaging data, the Oxford Centre for Functional Magnetic Resonance Imaging of the Brain (FMRIB) Software Library (FSL; www.fmrib.ox.ac.uk/fsl) was used. For correction about head motion effects and image distortions due to eddy currents, affine multi-scale two-dimensional registration was used. Fiber tracking was performed using a probabilistic tractography method based on a multifiber model, and applied using tractography routines implemented in FMRIB Diffusion (5000 streamline samples, 0.5 mm step length, curvature threshold = 0.2). The auditory radiation was identified by selection of fibers passing through both regions of interest (ROIs). The seed ROI was placed on the medial geniculate body of the thalamus and the target ROI was placed on the primary auditory cortex on an axial slice [20,22]. On DTT, the auditory radiation showed severe narrowing and tearing in both hemispheres (Figure 1).

## 3. Discussion

In this case study, on DTT for auditory radiation, we found severe narrowing and tearing in both hemispheres in a patient with tinnitus following whiplash injury. Severe narrowing and tearing of both the auditory radiations on DTT indicates neural injuries of both auditory radiations. Because no definite brain lesion was detected on conventional brain MRI, traumatic axonal injury (TAI) appeared to be the most likely pathogenetic mechanism [23,24,25,26]. We think that these injuries of the auditory radiations might be at least partly related to the tinnitus in this patient. On the other hand, the delayed onset (three days after the car crash) and aggravation with passage of time of the tinnitus in this patient suggest that the TAI was mainly ascribed to a secondary injury; this refers to a condition in which axons are not damaged at the time of injury, but undergo axonal injury caused by the sequential process of impaired axoplasmic transport, continued axonal swelling, and subsequent disconnection, to ultimate disconnection rather than a primary injury [24,27,28].

Several studies have suggested that tinnitus is related to neural injury of the central auditory pathways [8,20,29,30,31,32]. Regarding the tinnitus in patients with head trauma, Nolle et al. (2004) investigated the correlation between clinical symptoms of auditory dysfunction (tinnitus, hyperacusis, and hearing loss) and the audiological test results in 31 patients with blunt (acceleration/deceleration) head trauma [9]. The results of testing the central auditory pathway showed that the transiently evoked otoacoustic emissions revealed significant differences between amplitude differences of all patients as well as patients with tinnitus and controls. A complete loss of stapedial reflex responses was found in 12 patients and a partial loss in four patients. Auditory brainstem responses were normal in all patients, but 76% had lowered loudness discomfort levels. Therefore, they concluded that blunt trauma of the head can lead to auditory dysfunction, probably as a result of diffuse axonal injury of the central auditory pathway which is consistent with our results. Recently, Jang et al. (2019) reported on a case of auditory radiation injury with sensorineural hearing loss following mild traumatic brain injury [20]. To the best of our knowledge, this is the first study to demonstrate injury of the auditory radiation in a patient with tinnitus following whiplash injury. However, several limitations of this study should be considered. First, we could not employ high angular resolution DTI technique for reconstruction of the auditory radiation [22]. Second, the fact that we did not confirm the presence of micro-hemorrhagic lesions of TAI by using gradient recalled echo (GRE) or susceptibility-weighted imaging (SWI) of the brain in this patient is a further limitation of this study [33]. Third, we could not evaluate other central auditory pathways except for the auditory radiation because the reconstruction method for other central auditory pathways using DTT has not been reported so far. Fourth, because this is a case report, the study has limited generalizability. Therefore, additional prospective studies that include larger numbers of similar cases are warranted.

As a conclusion, neural injury of the auditory radiation was demonstrated in a patient with tinnitus following whiplash injury, using DTT. DTT might be helpful for diagnosis of tinnitus due to auditory radiation injury following TBI. Therefore, we suggest evaluation of auditory radiation using DTT for patients with tinnitus after TBI who show negative findings on conventional brain MRI. In addition, we think that a similar method using DTT could be applied for the diagnosis of TAI of other neural tracts.
